# Lumbar position sense and the risk of low back injuries in college athletes: a prospective cohort study

**DOI:** 10.1186/1471-2474-8-129

**Published:** 2007-12-31

**Authors:** Sheri P Silfies, Jacek Cholewicki, N Peter Reeves, Hunter S Greene

**Affiliations:** 1 Rehabilitation Sciences Research Laboratory, Drexel University, Philadelphia, PA, USA; 2 Biomechanics Research Laboratory, Department of Orthopaedics and Rehabilitation and Department of Biomedical Engineering, Yale University School of Medicine, New Haven, CT 06520, USA; 3 Northern California Orthopedic Centers, Carmichael, CA 95608, USA

## Abstract

**Background:**

Impaired proprioception in the lumbar spine has often been reported in people with low back pain. However, no prospective studies exist to assert the cause and effect of this association. We hypothesized that athletes with a history of low back injury (LBI) would demonstrate poorer lumbar position sense (PS) than athletes without a history of LBI, and that this deficit would be a risk factor for future LBI.

**Methods:**

This was a prospective cohort study with 2–3 year follow-up. Lumbar spine PS in the transverse plane was evaluated in 292 athletes using three tests: 1) passive and 2) active trunk repositioning, and 3) motion perception threshold. Mean absolute (accuracy) and variable (precision) errors were computed.

**Results:**

There were no significant differences in the repositioning errors or motion perception threshold between athletes with and without a history of LBI or between those who did and did not get injured during the follow-up. Active trunk repositioning resulted in smaller errors than passive repositioning (1.6°± 0.8°) versus 2.1°± 1.0°) and 1.7°± 0.8°) versus 2.3°± 1.1°) for the absolute and relative errors, respectively).

**Conclusion:**

Poor trunk PS in transverse plane is not associated with LBI in athletes, nor does it appear that poor trunk PS predisposes athletes to LBI.

## Background

Intact proprioception is essential for movement control [1-3]. In the spine, proprioceptive information is provided by structures present in the spinal ligaments, facet joints, intervertebral discs [4-6], and paraspinal muscles [7,8]. Muscle spindle density is high in deep paraspinal rotators, which are small muscles spanning one or two segments of the spine [9]. It is believed that the spindles in these muscles act as kinesthetic sensors that monitor trunk position and movement. It is these muscle receptors that are more likely responsible for information in the midrange of trunk motions [8,10]. While joint receptors cannot be discounted, these structures are thought to provide more input toward the end range of joint positions [10]. However, altered joint afferent information can alter muscle activation [11]. Consequently proprioceptive information from both muscle and joint receptors may be an important aspect of trunk control of motion. Since the overwhelming majority of low back injuries (LBI) in athletes are classified as soft tissue injury, the mechanoreceptors embedded in these tissues could be involved [12-14].

Proprioceptive impairments, reflected by poor joint position sense (PS), have been identified in numerous soft tissue injuries commonly suffered by athletes: anterior cruciate ligament deficiency [15,16], ankle sprains [17], glenohumeral instability [18,19], neck injury [20,21] and low back pain (LBP) [8,22-27]. However, in the LBP literature, the evidence regarding the presence of proprioceptive impairments is not unanimous. Newcomer et al. (2000) [28] reported significantly larger repositioning error in patients with LBP in trunk flexion and significantly lower error in trunk extension when compared to a control group. Field et al. (1991) [29] found less variability in repositioning error in their LBP group and Parkhurst et al. found no correlation between directly measured proprioceptive variables and LBI, but instead reported its association with the asymmetry indices derived from these variables [30]. Finally, several studies demonstrated no proprioceptive impairments in individuals reporting LBP or injury [31-34]. Differences in test conditions (body position, planes of motion, whether or not vestibular system is involved, lower body constraint), and subject characteristics could explain some of the divergent results in the literature.

Despite the above uncertainties, widely reported deficits in postural control [35-39] and altered patterns of muscle response to sudden trunk loading [40-42] among patients with LBP are hypothesized to be, at least in part, the result of injury to mechanoreceptors embedded in the soft tissues surrounding the lumbar spine. However, an alternative hypothesis would be that impaired spinal proprioception is a pre-existing risk factor that predisposes individuals to LBI.

The aims of this prospective study were to 1) examine whether differences in trunk PS exist between individuals with and without a history of LBI, and 2) determine if impairment in trunk PS results from injury or alternatively predisposes athletes to future LBI. Knowledge gained regarding trunk proprioceptive deficits would assist in developing targeted interventions for athletes. We hypothesized that athletes with a history of LBI would demonstrate less accurate and precise trunk repositioning and higher motion perception thresholds. Additionally, athletes with poor trunk PS would have a higher risk of sustaining a LBI than athletes with more accurate and precise trunk PS.

## Methods

### Subjects

Two hundred and ninety two Yale University athletes from 22 sports were recruited to participate in this study. The athletes represented a homogenous group with respect to age, general health and fitness level. All but 4 subjects were varsity athletes. Two of the 4 subjects participated in club level sports (rugby, martial arts), 1 in weight lifting and 1 was a nationally ranked badminton player. All provided written informed consent as approved by the Yale University's Human Investigations Committee. Prior to experimental testing, subjects completed a questionnaire containing personal data (age, height, weight, sport, years of participation at varsity level, past medical history), a 10-cm visual analog pain scale (0–100) [43], Roland Morris Disability Scale (0–24) [44], and additional questions regarding any previous LBI and recovery.

Incidence of LBI was recorded during a 2–3 years follow-up period. It varied slightly for each athlete due to the time elapsed between the testing session and graduation from college. During the follow-up, participating athletes received regular electronic mailings to ascertain their LBI status and to insure a high capture rate of LBI. Our operational definition of an injury was any LBP that caused the athlete to seek medical attention (physician, athletic trainer, or physical therapist) and to miss at least 3 days of participating in their sport or training routine. All inclusion and exclusion criteria were based on self-reported data, which were verified with training room and team physician records.

### Procedures

**Figure 1:**
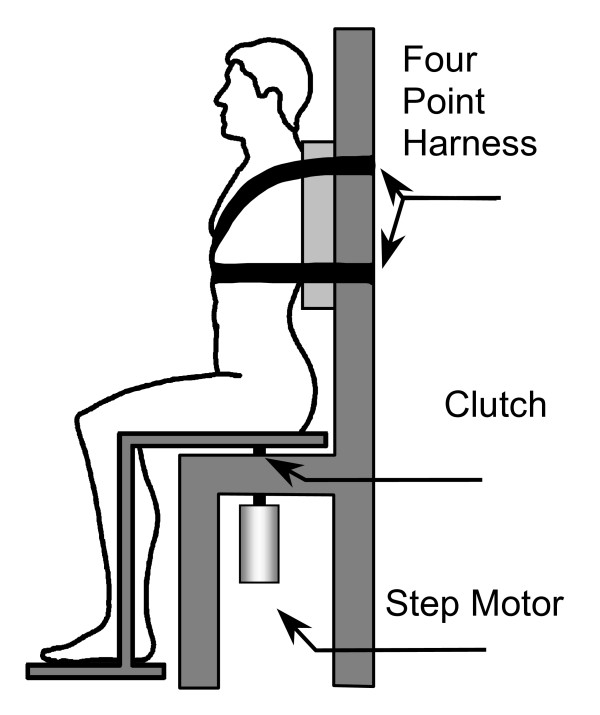
Apparatus. A subject positioned in the trunk position sense testing apparatus, such that a pivot axis coincided with the imaginary line drawn between the apex of the iliac crest and greater trochanter. The upper body was fixed to the backrest with a 4-point harness.

Lumbar spine PS in the transverse was evaluated using a specially built apparatus similar to the one used by Taimela et al. [25] (Fig. 1). It was designed to produce passive motion of the lumbar spine in the transverse plane. The resolution of the angular measurement of this apparatus was less than 0.01° and the accuracy obtained from the calibration curve was 0.35°. Subjects were positioned in the apparatus so that the vertical pivot axis coincided with the imaginary line drawn between the apex of the iliac crest and greater trochanter. Their arms were crossed with hands resting on opposite shoulders to eliminate cueing of lower body movement. The subject's legs rested on the feet support, creating a 90° knee angle. The seat was driven by a stepper motor at a steady slow rate to minimize tactile cueing. The contribution of the vestibular system was eliminated by securing the upper body to the backrest with a 4-point seatbelt. The subjects performed all trials with closed eyes. Auditory cues produced by the stepper motor were masked by background noise from a buzzer.

Three tests were performed: 1) passive and 2) active repositioning to neutral (zero degrees of trunk rotation) and 3) motion perception threshold (MPT). In all tests, subjects were given 2 practice trials in each direction and verbal feedback of their error. This was followed by 10 trials randomized for direction (5 trials in each direction). Subjects stayed in the apparatus between the tests and were coached not to change their seated position. None of the athletes reported any discomfort during testing.

The seat drive could be disengaged from the motor with a clutch to allow both passive (motor driven return) and active trunk repositioning tests. In either case, subjects were initially rotated 20° away from the neutral spine posture at 2.2°/sec and briefly held in that position (2.0 sec). In the passive test, subjects were then slowly rotated towards the original position by the stepper motor at 1.0°/sec [30]. In the active test, subjects rotated themselves back to neutral after the clutch was disengaged from the motor drive. In both tests, subjects recorded their perceived neutral position by pressing the hand held switch, which also stopped the apparatus.

The MPT test measured the smallest amount of rotation a subject could perceive. Starting in the neutral (zero) position, subjects were rotated either clockwise or counter-clockwise at a constant rate of 0.1°/sec. As soon as the motion was perceived, subjects stopped the rotation by pressing the switch and immediately stated the direction of movement. Subjects were returned to the neutral position following each trial. To avoid undesirable score variations from combining the MPT with directional motion perception, in only those trials, in which subjects correctly identified the direction of motion, was the degree of rotation recorded [30]. Testing continued until 5 data points were recorded for each direction of rotation.

### Data Analysis

Repositioning accuracy was the difference between neutral (0°) and the actual position the subject indicated as neutral. Using the mean absolute repositioning error, we found no significant differences between left and right rotations, so the data were combined to create ten measurements for each test. Of the 10 measurements, the 2 highest errors were eliminated to reduce variability and the mean of 8 measures was used for analysis. Two types of error were calculated for active and passive repositioning: absolute error (AE) and variable error (VE). AE is a measure of accuracy and represents the mean absolute value of the deviation between subjects' perceived neutral position and the actual neutral position without regard for direction of the error. The VE is a measure of precision and represents the average deviation of each trial from subjects' mean score. The MPT was the smallest amount of rotation from the neutral position that was perceived by the subject.

To address both hypotheses, a MANOVA was used for 2 repositioning parameters (AE and VE) with 3 between-subject factors 1) history versus no history of LBI, 2) injury versus no injury during the follow-up period, 3) gender, and 1 within-subject factor of test mode (passive versus active). When the MANOVA demonstrated a significant effect, univariate post-hoc tests were employed. A 3-way ANOVA was performed to determine group differences for MPT with 1) history versus no history of LBI, 2) injury versus no injury during the follow-up period, and 3) gender as the 3 factors. All statistical analyses were performed in Minitab (Minitab Inc., State College, PA).

All measures of trunk PS (active and passive AE, VE, and MPT) were examined for within-session repeatability using intra-class correlation coefficients (ICC) model (2, k) and standard error of measurement (SEM) [45]. For this purpose, the averages of the first five and the last five trials served as the test-retest values for all athletes.

## Results

Our results reflected 292 college athletes who could be undoubtedly classified as injured or not injured based on the availability of records and our definition of injury. Their characteristics are presented in Table 1. At the start of the study, 60 athletes (21%) had a history of LBI within the last five years. Majority of them (43(72%)) sustained only a single LBI episode whilst the remainder had multiple episodes. During the follow-up period, 31 athletes (11%) became injured (Table 1). Of these, 12 athletes (39%) had a history of LBI. The athletes injured during follow-up were significantly (p < 0.01) taller (1.80(0.09) m vs. 1.76(0.10) m) and heavier (78.9(15.3) kg vs. 72.0(12.4) kg) than the uninjured athletes. The effects of history and subject characteristics on LBI were addressed in a previous publication [46]. The current report focuses solely on trunk PS data.

**Table 1:** Characteristics of athletes with no history of low back injury (No Hx LBI) and with a history of low back injury (Hx LBI) at the start of study, and the athletes who sustained injury during the follow-up period (LBI during follow-up)*.

	Injury Status at the Start of Study (n = 292)				Injured During Follow-up	
	
	No Hx LBI (n = 232)		Hx LBI (n = 60)		LBI (n = 31)	
Gender	F	M	F	M	F	M

Number (n)	115	117	33	27	16	15
Age (yrs)	19.4 (1.0)	19.3 (1.3)	19.4 (1.0)	19.9 (3.0)	19.3 (0.9)	19.6 (1.2)
Height (cm)	169 (7)	183 (8)	172 (8)	183 (7)	174 (7)	186 (7)
Weight (kg)	64.9 (8.6)	79.7 (11.5)	67.4 (8.1)	82.7 (15.3)	69.5 (8.3)	88.8 (14.9)
Time Post Injury (months)†	--	--	24.0 (22.6)	21.0 (17.5)	--#	--#
VAS (0–100)‡	--	--	69.0 (14.1)	57.5 (22.2)	--#	--#
RMQ (0–24)§	--	--	5.5 (3.8)	5.2 (5.2)	--#	--#

*Data represent means (SD).

†Time Post Injury = number of months between the last LBI and testing.

‡VAS = visual analog pain scale (0–100) at time of injury (higher score means greater amount of pain).

§RMQ = Roland Morris Disability Questionnaire (0–24) at time of injury (higher score means greater level of disability).

#Subjects were not re-tested during the follow-up period.

**Figure 2:**
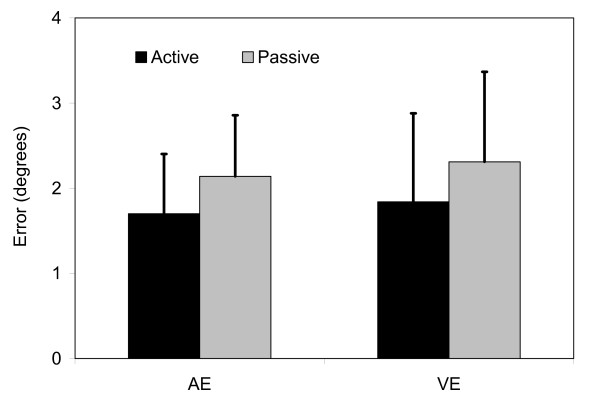
Significant differences existed between the active and passive measurements of trunk repositioning errors (p < 0.01). These differences were present in both average (AE) and variable (VE) errors. Data represents means with standard deviation bars (pooled across all trials and test modes).

**Figure 3:**
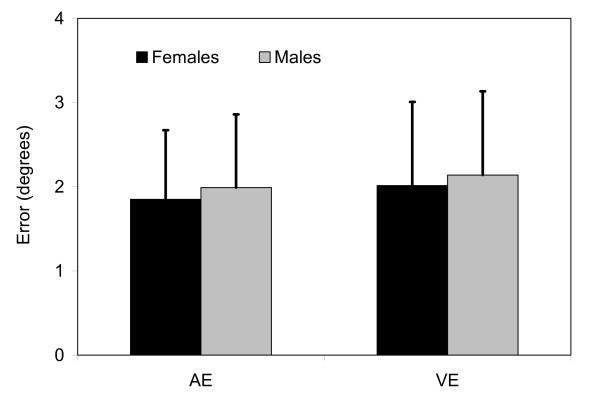
Significant differences existed in trunk repositioning accuracy between males and females (p = 0.04). These differences were present in both average (AE) and variable (VE) errors. Data represents means with standard deviation bars (pooled across all subjects and trials).

All data met assumptions of normality (Anderson-Darling test, Minitab, Inc.). The initial MANOVA returned no significant differences in trunk repositioning error in the transverse plane between the athletes with and without a history of LBI (p = 0.25) or between those who did and did not sustain a LBI during the follow-up period (p = 0.63) (Table 2). However, significant effects of test mode (passive or active) (p < 0.01) and gender (p = 0.04) were present. The post-hoc univariate analyses revealed that the athletes were significantly more accurate (AE, p < 0.01) and precise (VE, p < 0.01) in the active trunk repositioning tests as compared to the passive tests (Figure 2). Males were slightly (0.15°), but significantly less accurate (AE, p = 0.02) and less precise (VE, p = 0.01) than females (Figure 3).

**Table 2:** Average (AE) and variable (VE) trunk repositioning errors in the active and passive tests for athletes with or without history of low back injury (No/Hx LBI), and for athletes who did or did not sustained injury during the follow-up period (No/LBI)*.

	Active AE		Passive AE		Active VE		Passive VE	
	
	No LBI	LBI	No LBI	LBI	No LBI	LBI	No LBI	LBI
No Hx LBI	1.6 (0.8)	1.7 (0.7)	2.1 (1.0)	2.1 (0.7)	1.7 (0.8)	1.8 (0.7)	2.3 (1.0)	2.3 (0.8)
Hx LBI	1.6 (0.6)	1.4 (0.7)	2.2 (1.1)	2.2 (1.4)	1.7 (0.6)	1.5 (0.8)	2.4 (1.1)	2.3 (1.3)

*Data represent means (SD).

There were no significant effects of any of the factors on MPT. On average, all athletes perceived their trunk rotation at 1.1° (SD = 1.0°).

The within-session reproducibility of the active and passive repositioning tests was good (0.47 < ICC < 0.61, 0.57° < SEM < 0.73°, Table 3). The reproducibility of MPT was excellent with ICC = 0.89 and SEM = 0.34° (Table 3).

**Table 3:** Within-session repeatability* of average (AE) and variable (VE) trunk repositioning errors in the active and passive tests and motion perception threshold (MPT) measures.

	**ICC (2, k)**	**SEM (deg)**
Active AE	0.61	0.57
Passive AE	0.58	0.73
Active VE	0.47	0.58
Passive VE	0.59	0.69
MPT	0.89	0.34

*Repeatability was quantified with intra-class correlation coefficient (ICC) and standard error of measurement in degrees (SEM).

## Discussion

There is inconsistency in the literature with regards to impairment in trunk PS and LBP. Some studies have found impairment [8,22-27], whilst others have not [31-34]. Because of these inconsistencies, it was not possible to state objectively whether LBP was associated with impairment in trunk PS. To address this problem, we designed a large prospective study with a homogenous subject population using a similar protocol to that of Taimela, Leinonen, and colleagues [25-27]. This protocol has the advantage in that it isolates proprioception to trunk sensory receptors, while other inputs from lower extremities, vision, and the vestibular system are removed. Given the results of this study, we would conclude that impaired trunk PS is not associated with LBP. It appears that the majority of back injuries in athletes do not involve significant disruption of trunk PS, nor does it appear that poor trunk PS predisposes athletes to LBI. In comparison to previous studies, our results are strengthened by the use of a large homogenous subject group, measurement of several aspects of trunk proprioception, isolation of the trunk from lower extremity input and standardization of the range of movement of each subject around the neutral trunk position.

It is possible that our athlete population differs from the general population used in others studies with positive findings. Perhaps, factors such as age or fitness levels can account for the differences between the LBP and healthy controls. Proprioception declines with age [47,48] and more fit individuals may have more accurate joint PS. This notion has been supported by research demonstrating better knee MPT in trained gymnasts versus healthy non gymnasts [49].

It is also possible that other planes of motion can be affected more by LBP than the transverse plane used in our study. However, it should be noted that a number of the positive studies documented impairment in this plane [25-27]. So it would be expected that if impairment exists, it would also be found in the transverse plane of motion.

If impairment in trunk PS was strongly related to LBP, the findings in the literature would be more consistent. O'Sullivan *et al.*[23] suggested that the non-homogeneity of patients in terms of their specific pathologies may be responsible for the conflicting findings in research on trunk PS and LBP. The majority of injuries suffered by the athletes in our study were classified by health professionals in general terms as sprains or strains, and we did not attempt to diagnose these injuries further. It could be that impairment in trunk PS is specific to a particular patient population and/or pathology. This is still a possibility that needs to be investigated further.

It is unlikely that measurement limitations in our study could be responsible for the lack of differences in trunk PS between the athletes with and without a history of LBI or those who did and did not get injured during the follow-up. Our measurement errors (SEM) varied from 0.34° to 0.73° for the various test modes, and are in line with similar studies reporting diminished trunk PS in the LBP populations [25,30]. The magnitude of repositioning errors and the MPT obtained in the present study was also compatible with control groups in previous research (between 0.8° and 1.6°) [25,30]. More importantly, however, since our method was sufficiently sensitive to detect the differences in trunk repositioning accuracy between the active and passive testing modes (to be discussed shortly), it is likely that our data truly reflect the lack of impairment in athletes with a history of LBI. With similar confidence in our prospective study design, we can also reject the hypothesis that impaired trunk PS is a risk factor for sustaining a LBI in athletes.

Results from our study suggest differences between active and passive testing modes. Similarly to most of the relevant literature [50-56], we too found that active trunk repositioning resulted in smaller errors than passive trunk repositioning. It is generally agreed that afferent input from muscle spindle, encoding information tied to active movement, is in part responsible for a more accurate and precise joint PS in the active testing mode. However, other mechanisms, such as central corollary discharge, can be also used in a feedforward mechanism to assist in the reproduction of joint position [54].

Data from the current study revealed small gender differences in favor of females having slightly more accurate and precise trunk PS. However, clinical significance of differences in trunk repositioning error smaller than 0.15° is probably negligible. Thus, these findings should be interpreted more in line with other literature, which reported no gender differences [25,57].

## Conclusion

Many athletic rehabilitation programs emphasize proprioception training as it is believed that impaired joint PS may be a major risk factor for recurrent injuries [2,58]. While it is true that a history of LBI was the single best predictor of future LBI in athletes [14], based on our data, the mechanism mediating such injuries is not likely an impairment in trunk PS. Even in the present study, the athletes with a history of LBI had a 3-times greater risk of sustaining a LBI during the follow-up [46], but their trunk PS was not different from the athletes who had no history of LBI or those who did not sustain a LBI during the follow-up. If impairments in trunk PS exist during acute stages of LBI, they appear to recover relatively rapidly and do not constitute a risk factor for recurrent LBI.

## Competing interests

The author(s) declare that they have no competing interests.

## Authors' contributions

All of the authors were involved with data collection, analysis, interpretation of results, and manuscript preparation. In addition, JC designed the study and secured funding. All authors read and approved the final manuscript.

## Pre-publication history

The pre-publication history for this paper can be accessed here:


